# Data on intracellular localization of RPSA upon alteration of its redox state

**DOI:** 10.1016/j.dib.2015.12.017

**Published:** 2015-12-17

**Authors:** Filipe Vilas-Boas, Ana Bagulho, Ana Jerónimo, Rita Tenente, Carla Real

**Affiliations:** Centro de Química e Bioquímica, Faculdade de Ciências, Universidade de Lisboa, 1749-016 Lisboa, Portugal

**Keywords:** RPSA, RPS6, Redox signaling, Hydrogen peroxide, Ribosomes, Translation

## Abstract

Ribosomal Protein SA (RPSA), a component of the 40S ribosomal subunit, was identified as a H_2_O_2_ target in HeLa cells [1]. In order to analyze the intracellular localization of RPSA in different redox states we overexpressed wild-type RPSA (RPSAwt) or RPSA containing two cysteine to serine residue substitutions at positions 148 and 163 (RPSAmut) in HeLa cells. The transfected cells were exposed to H_2_O_2_ or N-acetylcysteine (NAC) and RPSA subcellular localization was assessed by immunofluorescence in permeabilized cells. In addition, co-immunofluorescence for RPSA and Ribosomal Protein S6 (RPS6) was performed in cells overexpressing RPSAwt or RPSAmut. Finally, the ribosomal expression of endogenous RPSA in the presence or absence of H_2_O_2_ was analyzed by Western blot.

The data presented in this work is related to the research article entitled “Hydrogen peroxide regulates cell adhesion through the redox sensor RPSA” [1].

**Specifications Table**

**Table:** 

Subject area	*Biology*
More specific subject area	*Redox signaling*
Type of data	*Images (microscopy and immunoblots), graph*
How data was acquired	*Immunofluorescence and image acquisition (Leica DMI 4000B inverted microscope with a spectral Leica TCS SPE confocal (Leica)), Western-blot and image acquisition (ImageQuant LAS 500 (GE Healthcare))*
Data format	*Figures containing confocal microscope processed images and analysis of Western blot by densitometry*
Experimental factors	*HeLa cell were cultured and transfected with pCIC-RPSAwt or pCIC-RPSAmut.*
Experimental features	*Transfected HeLa cells were incubated in control, oxidizing (with H*_*2*_*O*_*2*_*) or reducing (with N-acetylcysteine (NAC)) conditions. Untransfected cells were used to assess the expression of endogenous RPSA in ribosomes in oxidizing conditions.*
Data source location	*n.a.*
Data accessibility	*Data are supplied in this article*

**Value of the data**

• Ribosomal proteins are redox regulated.
• The redox state of ribosomal proteins might be relevant for their subcellular localization.
• The understanding of RPSA redox regulation could provide insights about new redox signaling mechanisms.
• This data can be used to study the role of H_2_O_2_ in RPSA-dependent translation.

## 1 Data

**Fig. 1:**
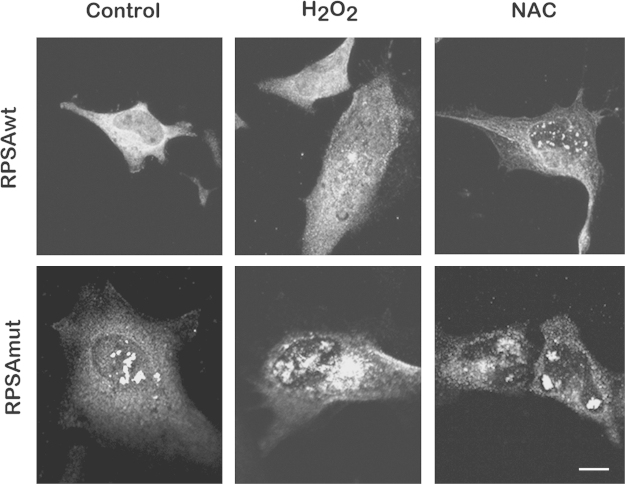
*RPSAwt or RPSAmut intracellular localization in different redox conditions.* HeLa cells were transfected with pCIC-RPSAwt or pCIC-RPSAmut and incubated in control, oxidizing (with H_2_O_2_) or reducing (with NAC) conditions. Maximum intensity projections of confocal images of immunofluorescence for RPSA in cells fixed with PFA and permeabilized with Triton X-100. Scale bar=10 µm.

HeLa cells were transfected with pCIC-RPSAwt or pCIC-RPSAmut and incubated in control, oxidizing (with H_2_O_2_) or reducing (with NAC) conditions. RPSA expression was then analyzed by immunofluorescence. The expression was cytoplasmic and nuclear (Fig. 1).

**Fig. 2:**
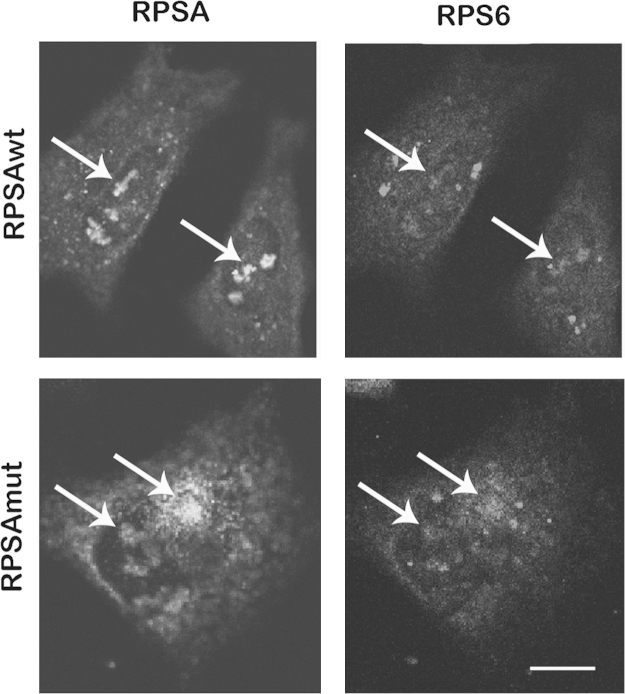
*Comparison of RPSAwt or RPSAmut intracellular expression with that of RPS6***.** HeLa cells were transfected with pCIC-RPSAwt or pCIC-RPSAmut. Single optical sections from confocal images of co-immunofluorescence for RPSA and the 40S ribosomal subunit RPS6 in cells fixed with methanol and permeabilized with Triton X-100. Arrows indicate regions of overlap. Scale bar=10 µm.

Overlap of RPSA and the 40S ribosomal subunit RPS6 was assessed by co-immunofluorescence in HeLa cells overexpressing RPSAwt or RPSAmut (Fig. 2).

**Fig. 3:**
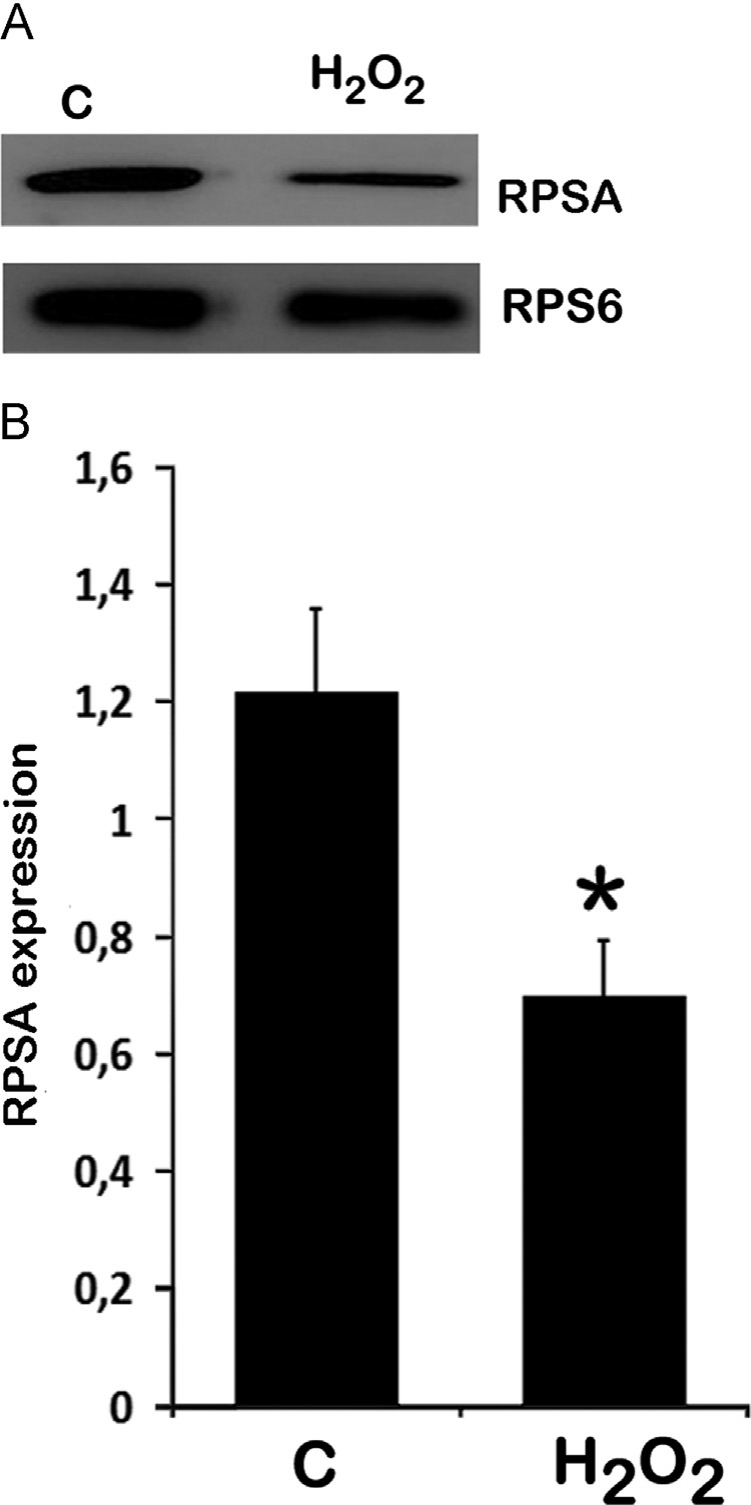
*Endogenous RPSA ribosomal expression in different redox conditions*. (A) Representative Western blot for endogenous RPSA expression in ribosomal extracts from HeLa cells, in control or oxidizing (with H_2_O_2_) conditions. RPS6 expression was used as loading control. (B) Quantification of Western blots (values show the mean and SEM; two-tailed Student’s *t* test, *p<0.02; *N*=3).

Western blot analysis of endogenous RPSA expression in ribosomal extracts was performed in HeLa cells in control or oxidizing (with H_2_O_2_) conditions (Fig. 3). H_2_O_2_ treatment decreased RPSA ribosomal expression.

## 2 Experimental design, materials and methods

### 2.1 Cell line, plasmids and cell transfection

HeLa cervical carcinoma cells were purchased from American Type Culture Collection (Manassas, VA, USA) and cultured in DMEM (Hyclone, Thermo Scientific) supplemented with 10% (*v*/*v*) fetal bovine serum (FBS, Sigma-Aldrich), penicillin-streptomycin solution (Hyclone, Thermo Scientific) and 2 mM l-glutamine (Hyclone, Thermo Scientific) in uncoated or 10 µg/ml laminin-coated plates (laminin, L2020, was purchased from Sigma-Aldrich). pCIC-RPSAwt (with the coding region of human RPSA [Uniprot:P08865]) and pCIC-RPSAmut (with the coding region of human RPSA containing two cysteine to serine residue substitutions at positions 148 and 163) are described in [1]. These plasmids were transfected into HeLa cells through lipofection with Lipofectamine 2000 (Invitrogen) according to manufacturer’s instructions. The presence of IRES:mCherry-NLS [2] in all plasmids allowed the identification of transfected cells.

### 2.2 Immunofluorescence and imaging

HeLa cells were plated on laminin-coated glass coverslips and left to adhere for 24 h, after which they were transfected with either pCIC-RPSA or pCIC-RPSAmut and incubated overnight. Cells were then incubated with 20–40 µM H_2_O_2_ by the steady state delivery method [3] for 30 min, after which they were fixed in 4% (w/v) PFA in PBS for 15–20 min at room temperature or in 100% methanol for 15 min at −20 °C. After fixation, cells were permeabilized with 0.5% (v/v) Triton X-100 in PBS for 15 min. Primary antibodies were then incubated overnight at 4 °C after blocking with 3% (v/v) FBS in PBS. Secondary antibodies were incubated for 2 h at room temperature. Immunofluorescence to detect wild-type or mutant RPSA was performed on fixed HeLa cells using the H-141 primary rabbit polyclonal antibody (1:100, sc-20979, Santa Cruz Biotechnology), or the F-18 primary goat polyclonal antibody (1:50, sc-21534, Santa Cruz Biotechnology) in the co-immunofluorescence experiment with the rabbit polyclonal α-S6 Ribosomal Protein (1:50, #2212, Cell Signaling). Secondary antibodies used were goat α-rabbit Alexa 488 (1:1000, Molecular Probes, Invitrogen); donkey α-goat Alexa 488 (1:1000, Molecular Probes, Invitrogen); goat α-rabbit Alexa 568 (1:1000, Molecular Probes, Invitrogen). Immunofluorescence images were obtained on a Leica DMI 4000B inverted microscope using a spectral Leica TCS SPE confocal. For this, we took Z-stacks for each of at least 5 independent fields per coverslip with a Leica ACS APO 63x/1.15 water objective, a step number of 5–20 and step size of 0.5–1 µm. Wavelength selection bandwidths were 500–560 nm for the 488 nm laser and 585–650 nm for the 532 nm laser.

### 2.3 Extraction of ribosomal proteins and Western blot

HeLa cells were plated in uncoated plates and incubated with a sublethal concentration of 20–50 µM H_2_O_2_ for 30 min using the steady state delivery method [3]. After H_2_O_2_ incubation, cells were harvested and ribosomal protein extraction was performed as described previously [4]. Briefly, cells were washed twice with ice-cold PBS and scraped into ice-cold PBS pH 7.4 and centrifuged for 5 min at 500 g. The pellet was resuspended in buffer A (250 mM sucrose, 250 mM KCl, 5 mM MgCl_2_, 50 mM Tris-HCl pH 7.4) followed by NP40 addition for a final concentration of 0.7% (v/v). Cellular suspension was incubated on ice for 10 min and centrifuged for 10 min at 750 g. The supernatant corresponding to the cytoplasmic fraction was further centrifuged at 12500 g for 10 min to eliminate the mitochondria present in the pellet. KCl was added to the supernatant to a final concentration of 0.5 M and centrifuged over a sucrose cushion (1 M sucrose, 0.5 M KCl, 5 mM MgCl_2_, 50 mM Tris-HCl pH 7.4) for 2 h at 250,000 g at 4 °C. The pellet containing the ribosomes was washed twice with ice-cold water and resuspended in a buffer containing 25 mM KCl, 0.5 mM MgCl_2_ and 50 mM Tris-HCl pH 7.4.

Protein loading buffer was added to each sample and protein content was analyzed by SDS-PAGE using 8% (w/v) polyacrylamide gels, after which it was transferred to a nitrocellulose membrane. Primary antibodies were diluted in blocking solution (PBS, 0.1% (v/v) Tween-20, 3% (w/v) milk) and added to the membrane which was incubated overnight at 4 °C. Primary antibodies used were goat polyclonal α-RPSA (F-18) (1:200, sc-21534, Santa Cruz Biotechnology) and rabbit polyclonal α-S6 Ribosomal Protein (1:200, #2212, Cell Signaling). Membranes were then washed and incubated with species-matched peroxidase-conjugated secondary antibody diluted in blocking solution (1:5000, sc-2020 and sc-2004, respectively, Santa Cruz Biotechnology). Labeled bands from washed membranes were detected by ECL (Millipore), images were acquired with ImageQuant LAS 500 (GE Healthcare) and protein expression was quantified with FiJi [5]. RPSA ribosomal expression was normalized by total protein loaded (Ponceau staining) (3 independent experiments).

## Conflict of interest

none
